# Effectiveness and tolerability of liraglutide as add‐on treatment in patients with obesity and high‐frequency or chronic migraine: A prospective pilot study

**DOI:** 10.1111/head.14991

**Published:** 2025-06-17

**Authors:** Simone Braca, Cinzia Valeria Russo, Antonio Stornaiuolo, Gennaro Cretella, Angelo Miele, Caterina Giannini, Roberto De Simone

**Affiliations:** ^1^ Department of Neurological Sciences, Reproductive Sciences and Odontostomatology University of Naples “Federico II” Naples Italy

**Keywords:** chronic migraine, glucagon‐like‐peptide‐1 receptor, intracranial pressure, liraglutide, migraine, calcitonin gene‐related peptide

## Abstract

**Objective:**

To assess whether glucagon‐like‐peptide‐1 receptor (GLP‐1R) agonists could serve as a novel prophylactic treatment for migraine in patients with obesity.

**Background:**

Increased intracranial pressure (ICP) is speculated to play a role in migraine mechanisms, as chronic migraine and idiopathic intracranial hypertension without papilledema (IIWHOP) are often clinically indistinguishable. These striking similarities suggest a deep pathogenetic link between the two conditions posing the hypothesis that control of ICP may be helpful in migraine treatment. The GLP‐1R agonists have been shown to greatly reduce ICP. Notably, they have also been demonstrated to decrease calcitonin gene‐related peptide expression in chronic migraine models.

**Methods:**

This was a prospective, interventional, open‐label, pilot cohort study, evaluating the effectiveness of liraglutide as an add‐on treatment of unresponsive migraine in patients with obesity. We consecutively enrolled patients with high‐frequency or chronic migraine and a body mass index (BMI) of >30 kg/m^2^, and unresponsive to at least two preventive treatments. We excluded patients with papilledema, sixth nerve palsy, or pulsatile tinnitus, to rule out patients in which idiopathic intracranial hypertension could be clinically suspected. Liraglutide was administered 1.2 mg daily. The study was conducted from January to July 2024, with a 12‐week follow‐up period. The primary outcome of this study was the reduction of monthly days with headache after 12 weeks of treatment with liraglutide compared to baseline.

**Results:**

We enrolled 31 patients (26 females, five males, with a mean [standard deviation, SD] age of 44.9 [14.6] years). The mean (SD) monthly days with headache decreased from 19.8 (6.7) to 10.7 (7.7) days post‐treatment. This change was significant with a mean difference of 9.1 days (95% confidence interval [CI] 5.41–12.84, *p* < 0.001, Cohen's *d*: 0.90). Conversely, BMI decreased slightly from a mean (SD) of 34.0 (2.3) to 33.9 (2.3) kg/m^2^, and this change was not significant (mean difference = 0.1 kg/m^2^, 95% CI −0.004 to 0.153 kg/m^2^, *p* = 0.060, Cohen's *d*: 0.34). Analysis of covariance indicated that age, sex, and concomitant medications did not significantly influence headache frequency reduction (all *p* > 0.050). Simple linear regression analysis showed that BMI reduction did not significantly predict headache frequency reduction (*β* = −1.448, 95% CI −19.390 to 16.495, *p* = 0.870, *R* ^2^ = 0.001), indicating no meaningful relationship between the two variables.

**Conclusion:**

Our findings show that liraglutide may be effective in the treatment of unresponsive high‐frequency or chronic migraine in patients with obesity, and that this effect is independent from weight loss. Although further studies are needed to clarify this topic, these findings generate the hypothesis that a derangement in ICP control may play a role in migraine pathogenesis and potentially represent a novel therapeutic target.

## Plain Language Summary

Obesity can worsen migraine by increasing headache frequency and reducing response to standard preventive treatments. We tested whether liraglutide, a medication developed for diabetes and weight management, with also an intracranial pressure lowering effect, could help patients with obesity and migraine that  do not respond to other treatments; after 12 weeks, participants experienced fewer headache days. Notably, this effect was independent of weight loss, suggesting that liraglutide may operate via different mechanisms, and represent a promising new approach to migraine prevention.

**Abbreviations**
CGRP: calcitonin gene‐related peptide
CI: confidence interval
CSF: cerebrospinal fluid
GLP‐1R: glucagon‐like peptide‐1 receptor
ICP: intracranial pressure
IIH: idiopathic intracranial hypertension
IIHWOP: idiopathic intracranial hypertension without papilledema
MHDs: monthly headache days
MIDAS: Migraine Disability Assessment
SD: standard deviation

## INTRODUCTION

Migraine is a prevalent neurological disorder affecting ~14.7% of the global population, causing significant burden on individuals and society.^1^ Despite advancements in migraine treatment, a substantial number of patients still face an unmet need, especially when preventive drugs prove ineffective.^2^ As such, there is a growing interest in exploring novel therapeutic approaches for migraine prophylaxis.

It has been extensively speculated about increased intracranial pressure (ICP) as possibly involved in migraine mechanisms.^3^ Indeed, chronic migraine shares with idiopathic intracranial hypertension without papilledema (IIWHOP) many overlapping features, and they are often clinically undistinguishable.^4^, ^5^ Both conditions share common risk factors such as obesity, female sex,^6^ and sleep disturbances,^7^ exhibit increased blood levels of calcitonin gene‐related peptide (CGRP),^8^ and present with a high prevalence of venous sinus stenosis,^9^ a known radiological marker of increased ICP.^10^ Additionally, they also share common treatments such as topiramate, a well‐defined preventive migraine treatment that greatly reduces ICP,^11^ as well as CGRP‐receptor monoclonal antibodies.^12^, ^13^ Actually, a raised ICP is found in 10–86% of chronic migraine series,^14^, ^15^, ^16^, ^17^ and such patients exhibit a sudden and sometimes sustained remission after even a single lumbar puncture with cerebrospinal fluid (CSF) subtraction.^15^, ^16^, ^17^ These similarities suggest the existence of a deep pathogenetic link between the two conditions and pose the hypothesis that a milder or intermittent deranged ICP control, although not sufficient for the papilledema to develop, might be much more prevalent than currently believed^18^ and is involved in migraine mechanisms, with significant therapeutic implications.

One potential intriguing avenue of investigation lies in the use of glucagon‐like peptide‐1 receptor (GLP‐1R) agonists, which have been primarily prescribed for the treatment of type II diabetes and obesity. GLP‐1R agonists play a crucial role in controlling fluid secretion.^19^ Studies have demonstrated that GLP‐1R agonists reduce sodium reabsorption and encourage diuresis by affecting the renal proximal tubule.^20^ Importantly, GLP‐1Rs are also present in the choroid plexus, which is the main structure responsible for CSF secretion in the brain.^21^

Recently, it was demonstrated that GLP‐1R agonists can reduce ICP in a rodent in vivo model, by inhibiting Na^+^K^+^‐ATPase in the choroid plexus epithelial cells, which represents the rate‐limiting step of CSF secretion.^22^ Notably, the decrease in ICP was observed both in rats with elevated ICP and in rats with normal ICP, and it was of greater significance compared to drugs commonly used in idiopathic intracranial hypertension (IIH), such as acetazolamide, or topiramate. These findings were confirmed by a recent randomized clinical trial in which exenatide, a GLP‐1R agonist, was administered in patients with IIH with significant reduction of the ICP, and a marked improvement in headache frequency.^23^

Very interestingly, it has been recently observed that GLP‐1R agonists are in fact able to reduce CGRP expression in chronic migraine animal models, and to suppress central sensitization in the trigeminal nucleus caudalis.^24^, ^25^

Therefore, this was a hypothesis‐driven study exploring the potential of GLP‐1R agonists as a novel prophylactic treatment for migraine. The central hypothesis posits that if a deranged ICP control is the shared pathogenetic step between migraine and IIHWOP, responsible for their striking clinical and pathogenetic similarities, then GLP‐1R agonists should improve migraine frequency and intensity. The aim of this study was to evaluate the effectiveness and tolerability of liraglutide for the treatment of unresponsive migraine in patients with obesity.

## METHODS

### Study design

This was a prospective, interventional, open‐label pilot cohort study evaluating the effectiveness of liraglutide as an add‐on treatment in patients with obesity (defined as a body mass index [BMI] of >30 kg/m^2^) and unresponsive high‐frequency episodic or chronic migraine. The study was approved by the Campania 3 Ethics Committee (2525‐OSS‐LIRA‐OBMIG‐2024) and all patients gave written informed consent before any procedure linked to the study.

### Study population

We consecutively enrolled 31 patients with obesity and migraine seen at our tertiary Headache Centre, who were offered liraglutide prescription between January 2024 and July 2024. Inclusion criteria for the present study were: diagnosis of high‐frequency episodic migraine (≥8 headache days/month) or chronic migraine fulfilling the International Headache Society criteria; headache diary documenting unresponsiveness to at least two treatments with standard care drugs and/or anti‐CGRP monoclonal antibodies for at least 12 weeks per therapy; BMI of >30 kg/m^2^; age >18 years; availability to undertake treatment with GLP‐1R agonists. Exclusion criteria were a diagnosis of diabetes; severe kidney, liver, or cardiovascular disease; history of pancreatitis; gastroparesis; concomitant use of dipeptidyl peptidase IV‐inhibitors, other GLP‐1R agonists or insulin; pregnancy and breastfeeding; age >75 years. We also excluded patients with papilledema, sixth nerve palsy, or pulsatile tinnitus, to rule out patients in which IIH could be clinically suspected.

### Treatment protocol

Liraglutide (at an initial daily dose of 0.6 mg in the first week and subsequent daily doses of 1.2 mg) was administered subcutaneously according to manufacturer's recommendations. Patients were asked to continue their current preventive therapies, anti‐CGRP monoclonal antibodies included.

### Study assessments and data collection

The duration of follow up was 12 weeks. Demographics and detailed medical history were recorded at baseline. For the study duration, patients were routinely asked to complete a headache diary. The patient's identity was known only to the treating physician. During data collection, privacy was guaranteed by assigning a pseudonym to each patient. Mean monthly headache days (MHDs) were evaluated by reviewing standardized paper patient headache diaries at baseline, and after 12 weeks. We asked patients to complete a headache diary and to report any other possible adverse effect. We assessed the migraine‐related clinical burden with the Migraine Disability Assessment (MIDAS) at baseline, and after 12 weeks. We assessed BMI at baseline, and after 12 weeks. There were no missing data.

### Outcomes

The primary outcome of this study was the reduction of baseline MHDs after 12 weeks of continuous treatment with liraglutide. The secondary outcomes were the reduction of BMI, MIDAS score, and the rate of adverse events.

### Statistical analysis

This is the primary analysis of these data. Descriptive analysis is reported for baseline variables, including frequency and percentage for categorical variables, and mean and standard deviation (SD) for continuous variables. Normality of the distributions was assessed using the Shapiro–Wilk test. The reduction of headache frequency was evaluated using a paired *t*‐test. We additionally performed paired *t*‐tests to assess the reduction in MIDAS score and BMI. We utilized analysis of covariance to explore if the change in headache days was influenced by potential confounders, including age, sex, and concomitant medications. The dependent variable in this model was the change in headache frequency. Homogeneity of variance was checked using Levene's test. Linearity between covariates and the dependent variable (assessed via scatterplots), and normality of residuals (evaluated using histograms and Q–Q plots) were met. Finally, we conducted a simple linear regression analysis to assess if the changes in headache frequency could be due to BMI reduction. Statistical significance was set at a two‐sided *p* value of 0.05. No statistical power calculation was conducted prior to the study as this was intended as an exploratory analysis, and therefore sample size was based on our previous experience with this design. Statistical analysis was performed using the Statistical Package for the Social Sciences (SPSS), version 29.0 (IBM Corp., Armonk, NY, USA).

## RESULTS

### Study population and baseline characteristics

A total of 38 eligible patients were approached for participation. Seven patients did not accept GLP‐1R agonist therapy, and therefore a total of 31 patients were included in the study, with no patients lost to follow‐up, or discontinuing therapy. Baseline demographic and clinical characteristics of the patients are detailed in Table 1.

**TABLE 1:** Baseline and demographic characteristics, including sex, age, migraine type (episodic vs. chronic), concomitant medications, headache frequency, body mass index, and Migraine Disability Assessment score at baseline.

Characteristic	Value
Number of patients, *n*	31
Sex, *n* (%)	
Female	26 (84)
Male	5 (16)
Age, years, mean (SD)	44.9 (14.6)
Migraine frequency, *n* (%)	
Episodic	4 (13)
Chronic	27 (87)
Concomitant medications, *n* (%)	31 (100)
Standard of care (propranolol, amitriptyline, topiramate, flunarizine, alone/combination), *n* (%)	11 (36)
Anti‐CGRP monoclonal antibodies, *n* (%)	6 (19)
Standard of care + anti‐CGRP monoclonal antibody, *n* (%)	14 (45)
Baseline headache frequency, days/month, mean (SD)	19.8 (6.7)
Baseline BMI, kg/m^2^, mean (SD)	34.0 (2.3)
Baseline MIDAS score, mean (SD)	60.4 (15.3)

Abbreviations: BMI, body mass index; CGRP, calcitonin gene‐related peptide; MIDAS, Migraine Disability Assessment Scale; *n*, number of patients; SD, standard deviation.

### Reduction in headache frequency

**FIGURE 1:**
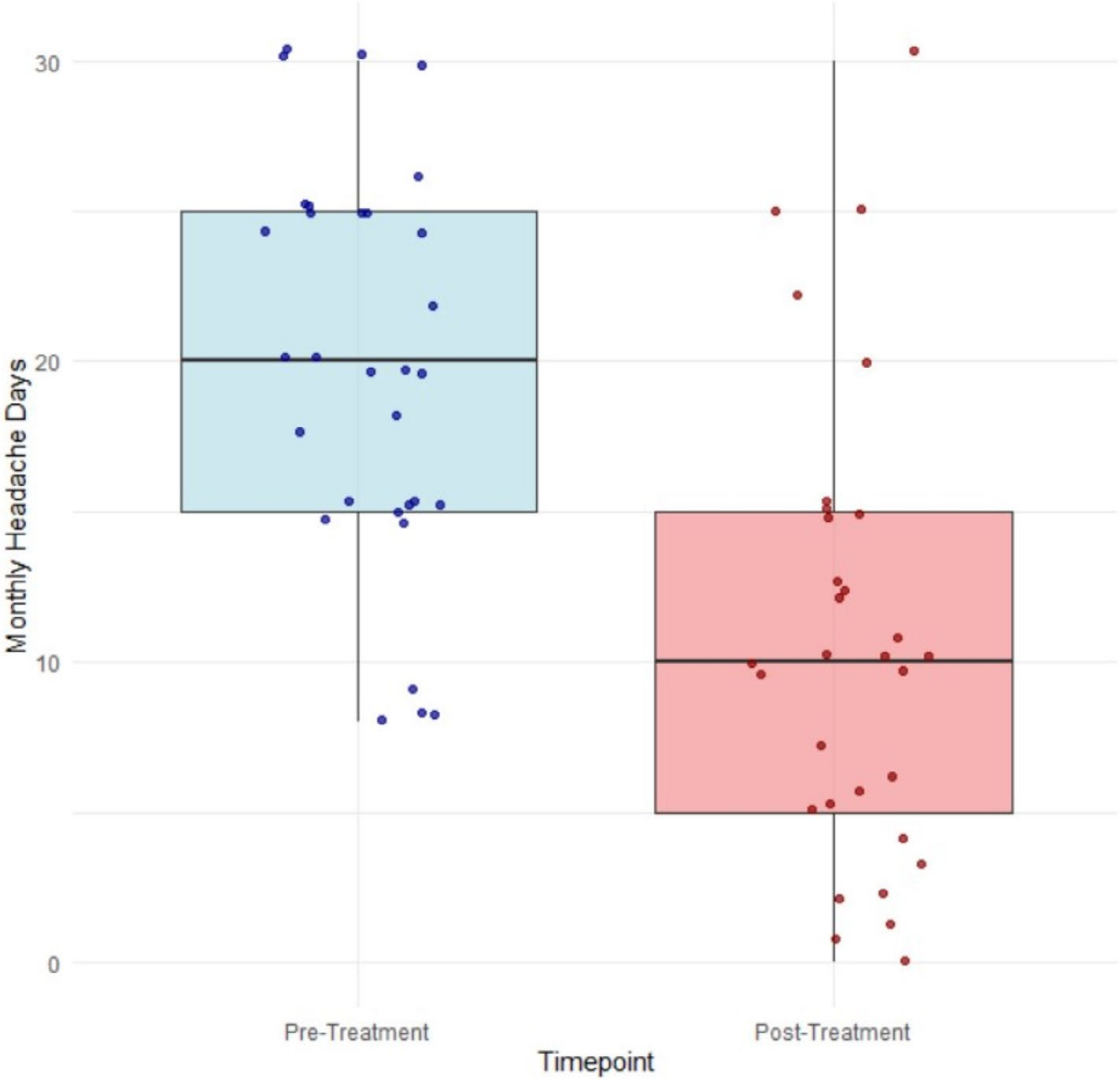
Mean monthly headache days. [Colour figure can be viewed at wileyonlinelibrary.com]

Roughly half of patients (*n* = 15 [48%]) had a ≥50% reduction in monthly headache frequency. A reduction ≥75% of MHDs was observed in seven (23%) patients and one patient (3%) showed complete resolution of the headache. The mean (SD) MHDs decreased from a baseline mean of 19.8 (6.7) to 10.7 (7.7) days post‐treatment. This change was statistically significant, with a mean difference of 9.1 days (95% confidence interval [CI] 5.41–12.84, *p* < 0.001, Cohen's *d*: 0.90) (Figure 1).

### Reduction in MIDAS score

**FIGURE 2:**
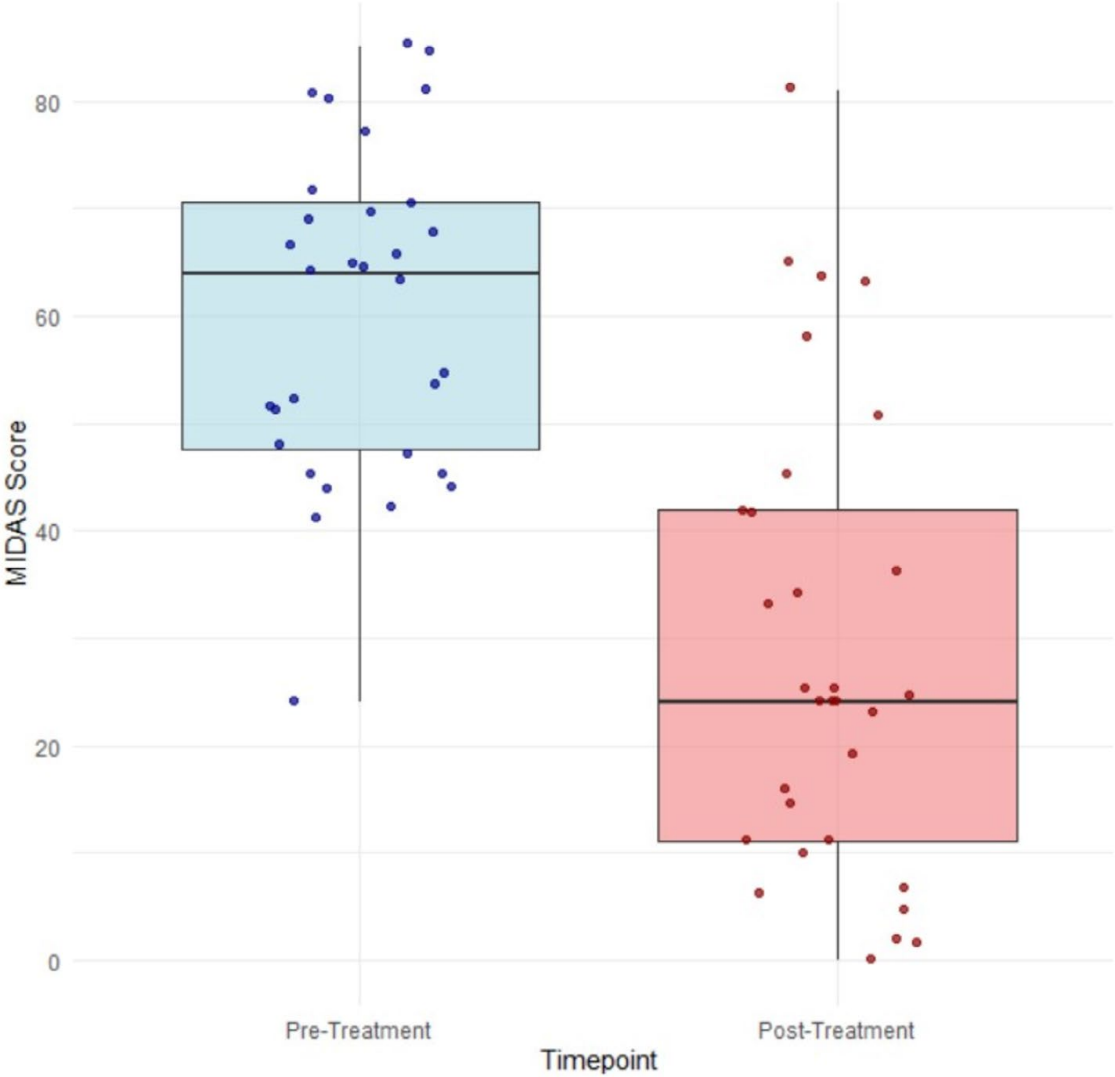
Mean Migraine Disability Assessment (MIDAS) score. [Colour figure can be viewed at wileyonlinelibrary.com]

The MIDAS score decreased from a baseline mean (SD) of 60.4 (15.3) to 28.6 (21.5) post‐treatment. This reduction was also significant, with a mean difference of 31.8 (95% CI 23.72–39.82, *p* < 0.001, Cohen's *d*: 1.45) (Figure 2).

### Changes in BMI

**FIGURE 3:**
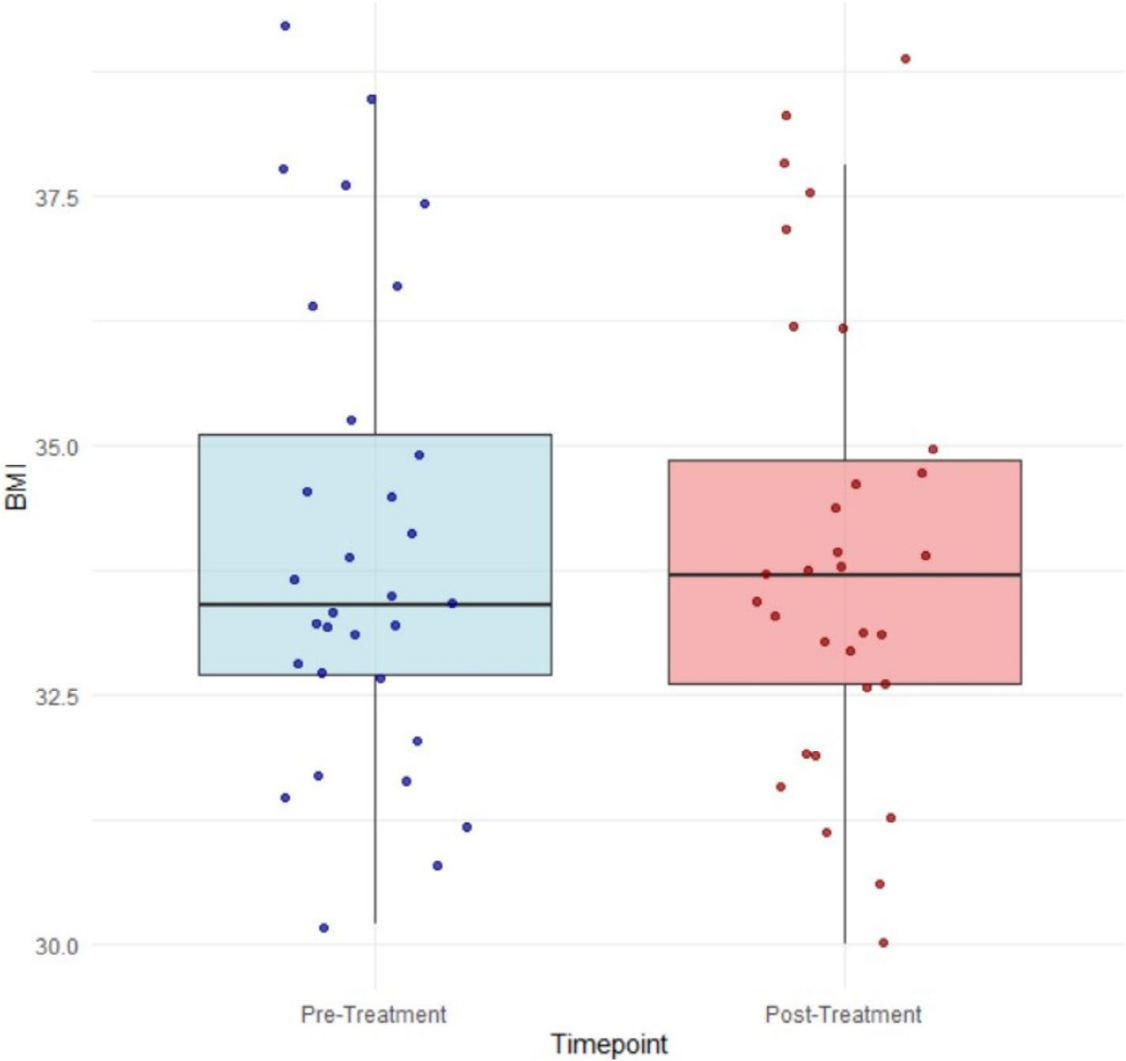
Mean body mass index (BMI). [Colour figure can be viewed at wileyonlinelibrary.com]

Conversely, BMI decreased slightly from a mean (SD) of 34.0 (2.3) to 33.9 (2.3) kg/m^2^, and this change was not statistically significant (mean difference = 0.1, 95% CI −0.004 to 0.153 kg/m^2^, *p* = 0.060, Cohen's *d*: 0.34) (Figure 3).

### Effects of covariates on headache frequency reduction

A repeated measures analysis of covariance was performed to assess whether age, sex, or concomitant medications influenced the change in headache frequency over time. Levene's test showed no significant violation of homogeneity of variance (*p* = 0.474). None of the covariates were found significant, regarding concomitant medications (*F* = 0.16, *p* = 0.696, *η* ^2^ = 0.01), age (*F* = 0.24, *p* = 0.629, *η* ^2^ = 0.01), and sex (*F* = 0.02, *p* = 0.895, *η* ^2^ = 0.00). These results suggest that the reduction in headache frequency was independent of age, sex, and concomitant medications.

### Regression analysis

A simple linear regression model assessed whether changes in BMI predicted changes in headache frequency. The analysis indicated no significant relationship between BMI change and headache frequency change (*β* = −1.448, 95% CI −19.390 to 16.495, *p* = 0.870), with an *R* ^2^ = 0.001, suggesting that BMI reduction explained only 0.1% of the variance in headache frequency reduction.

### Adverse events

We recorded mild adverse events in 13 (42%) patients, all of which were gastrointestinal in nature, (nausea, constipation); none of these events determined discontinuation of treatment, and they resolved spontaneously over the follow‐up period.

## DISCUSSION

This pilot study provides the first preliminary clinical evidence supporting the use of liraglutide, a GLP‐1R agonist, for the preventive treatment of migraine. We observed a significant reduction in the number of MHDs among patients receiving liraglutide, demonstrating that targeting the GLP‐1R may be an effective strategy for unresponsive migraine prevention. Conversely, we observed only a slight, non‐significant reduction in BMI, and regression analysis excluded any causal association between headache frequency and BMI reduction. This suggests that the therapeutic effect of GLP‐1R agonists on migraine observed in our study is independent of their weight loss effects, and that the mechanisms driving liraglutide's effectiveness in migraine prevention may operate independently of the significant metabolic effects GLP‐1R agonists have.

Recent evidence highlights GLP‐1R agonists as potent agents in reducing ICP, significantly more effectively than traditional treatments for intracranial hypertension, such as acetazolamide or topiramate, and consistent with our findings, this effect is unrelated to weight loss.^22^, ^23^

It is well‐established that elevated ICP is associated with increased dural sinus pressure and with dural sinuses compression.^26^, ^27^ Since the pioneering works of Ray and Wolf,^28^ it is known that the dural sinuses are the most pain sensitive structures of the brain.^29^ Notably, the trigeminal vascular innervation of dural sinuses is highly represented, and the main peptide expressed at this level is CGRP.^30^ Indeed, the stimulation of the dural venous sinuses has been shown to trigger the release of CGRP,^31^ and in conditions of altered ICP, blood studies have demonstrated elevated circulating CGRP levels.^13^ This activation of pain pathways can induce central sensitization, contributing to both the onset and the chronic progression of migraine.^18^, ^32^ Interestingly, migraine is indeed exacerbated by venous congestion,^33^ unlike other primary headaches.^34^

Several studies have demonstrated that reducing ICP can alleviate migraine symptoms. A recent study of 44 patients with chronic migraine who underwent lumbar puncture and CSF drainage observed a sudden improvement in headache frequency in 77.3% of the cases, maintained at 3 months in 45.4%, and at 12 months in 20.5% of the cases.^15^ Remarkably, this benefit was seen even in patients with opening pressures of <25 cm CSF, indicating that lowering ICP is effective even in the absence of defined IIH, aligning with similar reports in the literature.^16^, ^17^ This might imply that the reduction of intracranial compliance, that precedes the ICP raise, may be already pathological.^35^ Additionally, symptomatic treatment with mannitol, a potent ICP‐lowering agent,^36^ has been found particularly effective for status migrainosus,^37^ providing further evidence of the involvement of altered ICP in migraine pathophysiology. These findings further support the hypothesis that ICP reduction may represent a promising therapeutic approach for migraine.

By reducing ICP, GLP‐1R agonists might reduce CGRP release and central sensitization at the level of the trigeminal nucleus caudalis, contributing to their effectiveness in migraine prevention. If this finding was confirmed by randomized controlled trials on larger populations, GLP‐1R agonists might represent the first migraine treatment acting by preventing CGRP release rather than controlling its effects on pain pathways.

We acknowledge several limitations in this study. We did not measure ICP, fasting glucose levels, or hemoglobin A1c, so the exact mechanism of action of liraglutide remains speculative. There are some other potential explanations for liraglutide's impact on migraine, including shifts in sodium reabsorption, possible changes in Na^+^/K^+^ levels in the CSF, and improvements in glucose metabolism even in the absence of weight loss. Future research should evaluate these parameters to better elucidate liraglutide's mechanism of action in these patients. The lack of randomization and absence of a control group prevent us from definitively establishing a causal relationship between liraglutide and improvements in headache frequency. For this reason, although a history of multiple treatment failures may reduce the likelihood of a placebo response, it remains possible that the observed improvement could in part be due to placebo effect or regression to the mean. Future randomized controlled trials, with a comparator arm, are needed to confirm the effectiveness of GLP‐1R agonists in migraine prevention. Another limitation is the single‐center design, which may limit the generalizability of our findings. The study population was restricted to patients with obesity and unresponsive migraine, meaning that the results may not be extrapolated to non‐obese individuals, or those with better responses to standard migraine treatments. However, the use of real‐world data in a well‐characterized clinical population enhances the study's relevance for similar patient groups. Additionally, the follow‐up period of 12 weeks may not be sufficient to determine the long‐term effects of liraglutide on migraine frequency. While this duration allows for an assessment of early effectiveness, it remains unclear whether the observed benefit would be sustained over longer periods, whether tolerance may develop, or whether adverse effects might increase at higher doses or with prolonged use. Future studies, with extended follow‐up durations, and higher dose evaluations, are necessary to better understand the long‐term tolerability and effectiveness of liraglutide in migraine prevention. Finally, the small sample size limits statistical power. However, as an exploratory pilot study, these findings provide a foundation for larger‐scale trials aimed at further investigating the role of GLP‐1R agonists in migraine management.

## CONCLUSION

Our findings show that liraglutide may be effective in patients with obesity and high‐frequency or chronic unresponsive migraine. Notably, effectiveness seems to be independent from weight loss. Although the mechanisms responsible for liraglutide effectiveness in migraine prevention remains to be clarified, our finding generates the hypothesis that a derangement in ICP control may play a role in migraine pathogenesis, and potentially represent a new promising migraine therapeutic target. If confirmed by further randomized and controlled studies, liraglutide may represent the first migraine treatment aimed at preventing CGRP release rather than blocking its peripheral effects.

## AUTHOR CONTRIBUTIONS

**Simone Braca:** Conceptualization; data curation; formal analysis; investigation; supervision; validation; writing – original draft; writing – review and editing. **Cinzia Valeria Russo:** Investigation; writing – review and editing. **Antonio Stornaiuolo:** Investigation. **Gennaro Cretella:** Investigation. **Angelo Miele:** Investigation. **Caterina Giannini:** Investigation. **Roberto De Simone:** Conceptualization; methodology; project administration; supervision; validation; writing – review and editing.

## FUNDING INFORMATION

The authors received no financial support for the research, authorship, and/or publication of this article.

## CONFLICT OF INTEREST STATEMENT

**Simone Braca** and **Roberto De Simone** received personal compensation from Eli Lilly for oral presentations. **Cinzia Valeria Russo** received personal compensation from Sanofi Genzyme and Merck Serono. **Gennaro Cretella**, **Angelo Miele**, **Caterina Giannini**, and **Antonio Stornaiuolo** declare that there is no conflict of interest.
